# Cognitive control dysfunction and abnormal frontal cortex activation in stimulant drug users and their biological siblings

**DOI:** 10.1038/tp.2013.32

**Published:** 2013-05-14

**Authors:** D G Smith, P S Jones, E T Bullmore, T W Robbins, K D Ersche

**Affiliations:** 1 Behavioural and Clinical Neuroscience, Departments of Psychology and Psychiatry, University of Cambridge, Cambridge, UK; 2 GlaxoSmithKline, Clinical Unit Cambridge, Cambridge, UK; 3 Cambridgeshire and Peterborough NHS Foundation Trust, Cambridge, UK

**Keywords:** cognitive control, endophenotype, fMRI, inferior frontal gyrus, stimulant dependence, Stroop

## Abstract

Cognitive and neural abnormalities are known to accompany chronic drug abuse, with impairments in cognition and changes in cortical structure seen in stimulant-dependent individuals. However, premorbid differences have also been observed in the brains and behavior of individuals at risk for substance abuse, before they develop dependence. Endophenotype research has emerged as a useful method for assessing preclinical traits that may be risk factors for pathology by studying patient populations and their undiagnosed first-degree relatives. This study used the color-word Stroop task to assess executive functioning in stimulant-dependent individuals, their unaffected biological siblings and unrelated healthy control volunteers using a functional magnetic resonance imaging paradigm. Both the stimulant-dependent and sibling participants demonstrated impairments in cognitive control and processing speed on the task, registering significantly longer response latencies. However, the two groups generated very different neural responses, with the sibling participants exhibiting a significant decrease in activation in the inferior frontal gyrus compared with both stimulant-dependent individuals and control participants. Both target groups also demonstrated a decrease in hemispheric laterality throughout the task, exhibiting a disproportionate increase in right hemispheric activation, which was associated with their behavioral inefficiencies. These findings not only suggest a possible risk factor for stimulant abuse of poor inhibitory control and cortical inefficiency but they also demonstrate possible adaptations in the brains of stimulant users.

## Introduction

Disability in drug-dependent individuals is marked by impairments in cognitive function and concomitant neural biomarkers. Abnormalities in the orbitofrontal cortex and loss of prefrontal gray matter (GM) commonly accompany prolonged drug abuse,^1, 2, 3, 4, 5, 6^ along with increases in impulsivity and poor inhibitory control.^7, 8^ These deficits are typically viewed as the consequence of protracted stimulant use;^9^ however, evidence suggests that they may also predate heavy drug taking and facilitate the transition from recreational to compulsive use.^10, 11, 12, 13, 14^ Such behavioral and neurological traits may also serve as endophenotypes for dependence, predisposing for addiction.

The color-word Stroop is a well-known test of cognitive inhibition,^15, 16^ assessing executive control over an automatic behavior (word-reading) in favor of a more unusual behavior (color-naming). Interference represents a conflict between the two sources of information (font color and word meaning), and cognitive control is needed to overcome this conflict. Performance is measured by response latencies and interference scores, derived from the difference between congruent and incongruent trial response times (RTs). Greater discrepancy between these conditions indicates increased impairment.

Previous research investigating Stroop performance in stimulant-dependent individuals (SDIs) has shown impaired inhibition and corresponding abnormalities in brain activation.^17, 18, 19^ Performance has been used to successfully predict drug treatment outcomes,^20, 21, 22^ and activity in the prefrontal cortex (PFC), anterior cingulate cortex (ACC) and dorsal striatum correlated with self-reported abstinence and time spent in rehabilitation facilities.^19, 20^ Other studies of the Stroop have reported no differences in behavioral performance between SDIs and controls, but have shown significantly different activation in the orbitofrontal cortex, inferior frontal gyrus (IFG), ACC, parietal lobe, thalamus and caudate nucleus.^20, 23, 24, 25, 26, 27^

It is unknown whether these behavioral impairments and corresponding neural abnormalities are due to pre- or postmorbid factors—that is, whether they precede or are the result of long-term drug taking. One way to address this is to look for endophenotypes of SDIs. Endophenotypes are stable quantifiable variables (such as neurocognitive abilities) associated with genetic risk for a disorder and are abnormal both in patients and their relatives.^28, 29^ Comparing SDIs and their unaffected biological siblings could provide insight into whether the impairments seen in SDIs are precursory or consequential to drug abuse. Shared abnormalities in behavioral performance and brain activation during the color-word Stroop would suggest an endophenotype of impaired cognitive control and increased risk for stimulant dependence.

On an assessment of Stroop performance in SDIs, their biological siblings and unrelated healthy control volunteers, we predicted: (1) that both SDIs and their siblings would be impaired compared with controls, suggesting an endophenotype of poor cognitive control; (2) that SDIs would be more impaired than their siblings, indicating further drug-induced disability; and (3) that interference scores would correlate with abnormalities in IFG activation on the task, a region previously implicated in inhibitory control.^19, 30, 31^

## Materials and methods

### Participants

Recruitment and screening processes have been described in earlier work.^13, 14, 32^ Three equal groups of 50 SDIs, 50 of their non-dependent biological siblings and 50 unrelated healthy control volunteers were tested according to protocol approved by the Cambridge Research Ethics Committee. Participants were between the ages of 18 and 55 years, had no history of psychotic or neurodevelopmental disorder, neurological illness or traumatic head injury and were fluent in English. Participants were screened for mental illness using the Structured Clinical Interview for Diagnostic and Statistical Manual of Mental Disorders, 4th edition text revision Axis I Disorders (SCID),^33^ semistructured interview for drug history and checked for current physical health, color blindness and demographic information. Written informed consent was obtained before enrollment.

SDIs were included in the study if they satisfied Diagnostic and Statistical Manual of Mental Disorders, 4th edition text revision criteria for cocaine (94%) or amphetamine (6%) dependence, had a first-degree sibling who had no personal history of drug abuse (with the exception of nicotine), shared both biological parents and was able to take part. Control volunteers had no personal or family history of drug or alcohol dependence and were matched for age, gender and education levels. Severity of drug use in SDIs was measured using the Obsessive Compulsive Drug Use Scale (OCDUS),^34^ age of onset and years of use. Drug abuse tendencies in control and sibling participants were assessed using the Drug Abuse Screening Test (DAST-20).^35^ Alcohol use and depression levels were measured in all participants using the Alcohol Use Disorder Identification Test (AUDIT)^36^ and Beck Depression Inventory (BDI-II).^37^

Twelve individuals were excluded because of head movement (>1.5 mm in any direction, or enough to cause interslice variance), the presence of a clinically significant structural abnormality or inadequate task performance in which blocks of trials were discarded because of no correct responses. This resulted in 138 total participants (control=47; SDI=42; sibling=49).

### Measures

The color-word Stroop was administered during functional magnetic resonance imaging (fMRI) scanning. Participants were presented with one of four color words displayed in one of the four font colors. They were asked to identify the font color of the word using a four-button box, each button corresponding to a color. Participants were trained on button-color allocation before entering the scanner. During the congruent condition, the word was identical to the font color in which it was presented; for the incongruent condition, the word was displayed in one of the other three colors. Performance was measured by accuracy and response latencies. Interference scores were calculated by subtracting the difference in median RTs on correct trials between the challenge and control conditions (incongruent–congruent).

### Procedure

The task was administered as a blocked paradigm to prevent interfering carry-over effects between conditions.^19, 24^ Two blocks were presented containing 16 trials of either congruent or incongruent color words; the order of words within each block was randomized and the order of blocks was counterbalanced across participants. Each trial lasted 2.2 s, the stimulus word presented for 1.9 s, followed by an intertrial fixation cross for 0.3 s. The allocation of font colors to words was randomized; each of the four colors occurred equally often in each condition and no two identical colors ever followed one another. A cigarette break was allowed no less than 1 h before scan time to prevent either nicotine withdrawal or acute nicotine effects from influencing performance.

Whole-brain fMRI data were acquired at the Wolfson Brain Imaging Center, University of Cambridge (Cambridge, UK), using a Siemens Magnetom TIM Trio scanner operating 3 T (Siemens Medical Solutions, Erlangen, Germany). During the task, 32 transaxial sections of gradient-echo, echoplanar imaging data depicting blood oxygen level-dependent contrast were acquired parallel to the intercommissural line with the following parameters: repetition=2000 ms, echo time=30 ms, flip angle=78°, slice thickness=3 mm plus 0.75 mm, matrix of 64 × 64 with field of view=192 × 192 mm^2^ giving 3 × 3 mm^2^ in-plane resolution. Before data analysis, the first five images were discarded for T1 equilibration. T1 structural scans were collected using magnetization-prepared rapid acquisition gradient-echo sequence: 176 slices of 1 mm thickness, with TR (repetition time)=2300 ms, TE (echo delay time)=2.98 ms, TI (inversion time)=900 ms, flip angle=9° and field of view=240 × 256 mm^2^.

### Data analysis

Behavioral data were analyzed using Statistical Package for Social Science (SPSS v. 18, Chicago, IL, USA). Analysis of variance and *χ*^2^ tests assessed differences in demographic information. A general linear model (GLM) multivariate analysis in a 2 × 3 condition by group design with Bonferroni *post hoc* corrections was used to compare RTs, as well as interference scores and error rates between groups. Paired-samples *t*-tests assessed RTs within groups. Median scores were used in response latency analyses, and significance levels were set at *P*<0.05. In preparation for parametric analyses, BDI-II data were square-root transformed to reduce skew. As gender, education, smoking status, AUDIT and BDI scores differed between participant groups, multivariate analyses were conducted both with and without these variables as covariates.

FMRI analysis was conducted using Cambridge Brain Analysis software (CamBA; http://www.bmu.psychiatry.cam.ac.uk/software/). Data were preprocessed to correct for motion, differential slice-timing and smoothed in-plane by 0.5 voxels.^38, 39, 40^ Cluster significance levels were set for all imaging analyses using family-wise error correction for multiple comparisons *P*<0.05. Significant cluster values from CamBA fMRI contrast analyses were exported and further evaluated comparing group activation means using GLM multivariate analyses with Bonferroni corrections in SPSS. Correlations between behavioral data and group fMRI contrast activations were also conducted using Pearson's correlations in SPSS.

First-level whole-brain analysis measured activation among all participants contrasting incongruent–congruent conditions on successful trials. A design matrix composed of trial onset and RTs was convolved with hemodynamic response function,^41^ producing statistical maps of voxel-wise responses. These maps were normalized to Montreal Neurological Institute standard space by affine transformation to an echo-planar imaging template (http://www.fil.ion.ucl.ac.uk/spm) to obtain a group activation map of the contrast.

A three-way GLM omnibus analysis in CamBA assessed group differences in activation on the incongruent–congruent contrast. In accordance with previous studies with the Stroop,^20^ as well as our *a priori* hypothesis, this group analysis was repeated with restricted search volume masks of the IFG and ACC, taken from Hammer's probabilistic atlas.^42^

Behavioral interference scores were regressed onto first-level contrast clusters, and within-groups GLM was processed among all participants in CamBA. This resulted in a group activation map of significant clusters from the incongruent–congruent contrast that directly correlated with behavioral interference scores in all participants. This analysis was repeated with restriction to the IFG.

A GM voxel-based morphometry analysis previously conducted in these individuals^14^ was used, with *post hoc* application of the IFG mask to focus differences in cortical volume to this region. The voxel-based morphometry analysis was originally conducted using FSLVBM (http://www.fmrib.ox.ac.uk/fsl/fslvbm/index.html) on T1-weighted images collected during the same session as the functional data, and then compared for group differences using the CamBA software for permutation testing.^43^ See Ersche *et al.*^14^ for full details and imaging parameters. In this study, IFG GM volume was compared between groups using analysis of variance and correlated with behavioral performance using Pearson's coefficients.

## Results

SDIs had been on drugs for an average of 15.7 years (±6.4 s.d.), beginning use at the age of 16.5 (±2.9 s.d.). Ninety-three percent (*n*=39) tested positive for stimulants at the time of testing using urinalysis drug screen, with a mean time since last use of 2.2 days (±2.4 s.d.). SDIs had significantly higher depression and alcohol abuse scores than both sibling and control participants. There were also significantly more males in the stimulant-dependent group and higher rates of cigarette smoking (Table 1). Control and sibling participants did not differ in terms of sex, depression rates or alcohol use, although there were differences between the two groups in smoking status (see Supplementary Information for Bonferroni *post hoc* analyses of demographic data). There were no differences in age or IQ between any of the three groups, and only SDIs and controls differed in terms of educational attainment.

### Stroop performance

Using GLM multivariate analyses, significant differences arose between groups on response latencies for congruent (F(2,135)=7.243, *P*=0.001) and incongruent (F(2,135)=4.452, *P*=0.013) trials. These values remained significant after controlling for gender, education, smoking status, alcohol use and BDI depression scores in the model. As there were no differences in any of the results when using covariates or not, we report the rest of the results without covariates in the model. See Supplementary Information for a full set of results with covariates. In the congruent condition, SDIs had significantly slower responses than controls (Bonferroni *post hoc P*=0.001), while the siblings were slower than controls at *P*<0.1. On incongruent trials, both the SDIs (*P*=0.031) and their siblings (*P*=0.035) were significantly slower than controls (*P*=0.036). Stimulant-dependent and sibling groups did not differ from one another in either condition.

There were no differences between groups on interference scores contrasting congruent from incongruent trials (*F*(2,135)=0.797, *P*=0.45). However, in a paired-samples *t*-test all three groups were significantly slower on the incongruent than congruent condition within their own cohorts (controls: *t*(46)=7.319, *P*<0.001; SDI: *t*(41)=5.372, *P*<0.001; siblings: *t*(48)=7.327, *P*<0.001). This confirms the additional cognitive load of the incongruent trials, regardless of group. Groups did not differ in the number of errors made on the task (F(2,135)=1.915, *P*=0.151).

### Neuroimaging analysis

**Figure 1:**
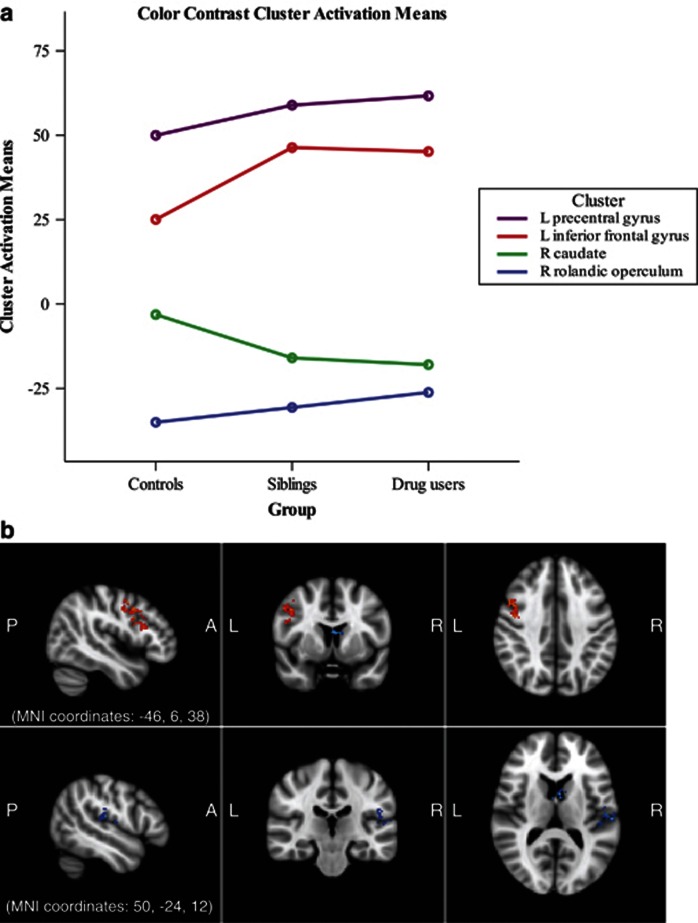
Whole-brain functional magnetic resonance imaging (fMRI) activation among stimulant-dependent individuals (SDIs) (*n*=42), siblings (*n*=49) and healthy controls (*n*=47) on the Stroop, contrasting congruent from incongruent color-word median response latencies. Four clusters were identified as having significantly different activation on incongruent than congruent conditions, with peak values in the left precentral gyrus (Montreal Neurological Institute coordinates (*x*, *y*, *z*) in millimeters: −44, 4, 34), left inferior frontal gyrus (−50, 14, 28), right caudate (4, 4, 12) and right rolandic operculum (60, −22, 12). Activation in these regions did not differ between groups. (**a**) Color contrast fMRI activation means by group. Analysis of covariance covariates include gender, smoking status, Alcohol Use Disorder Identification Test (AUDIT) and Beck Depression Inventory (BDI) scores. (**b**) fMRI group color contrast activations in the four significant clusters, with significant activation in the left inferior frontal and precentral gyrus, and significant deactivation in the right caudate and rolandic operculum on the incongruent compared with congruent color conditions.

In a first-level analysis, four clusters emerged that significantly differed in activation on incongruent–congruent contrast among all participants. Increases in activity on incongruent compared with congruent trials were seen in the left IFG, including the dorsolateral prefrontal cortex, and precentral/middle frontal gryus, while decreases in activation on the incongruent compared with congruent trials were present in the right rolandic operculum and caudate (Figure 1 and Table 2). These differences in blood oxygen level-dependent signal represent greater (or less) relative activation in all participants during performance of incongruent word trials, as compared with activity present during performance of congruent word trials. Follow-up analyses using a GLM multivariate analysis conducted in SPSS comparing group activation in these four clusters did not reveal any differences in activity levels between groups.

In a whole-brain omnibus group comparison, two clusters emerged that significantly differed between groups during the incongruent–congruent contrast: right insula/rolandic operculum (F(2,135)=18.373, *P*<0.001) and left medial/superior frontal gyrus (F(2,135)=12.094, *P*<0.001; Table 2). In these regions, siblings registered a significant relative decrease in activation compared with control and stimulant-dependent participants (Bonferroni *P*<0.001 for all comparisons). SDIs and controls did not differ from one another.

**Figure 2:**
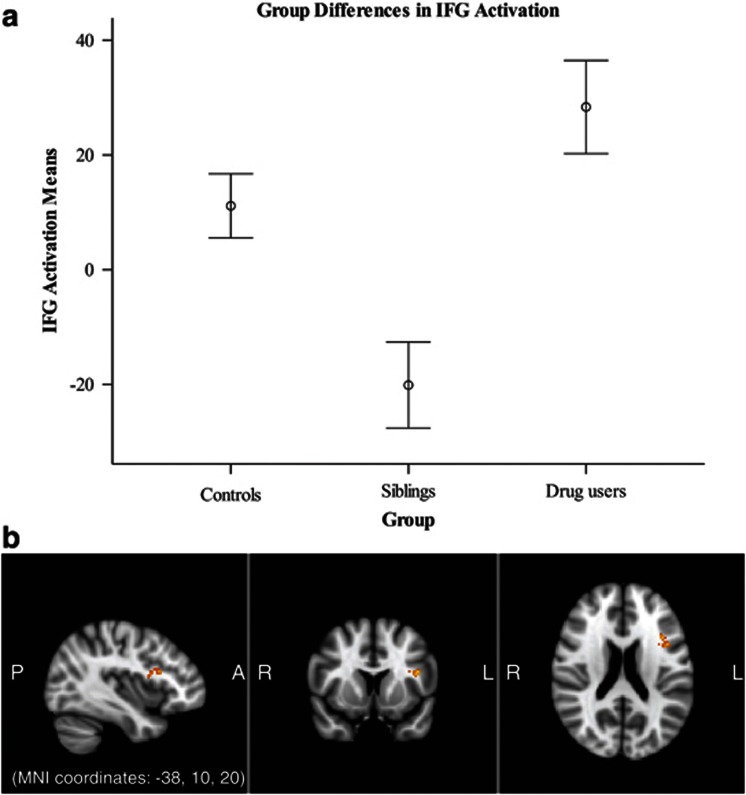
Activation differences between groups in the inferior frontal gyrus on the Stroop task upon application of a mask of the inferior frontal gyrus (IFG). Sibling participants (*n*=49) demonstrated a significant deactivation in the left IFG as compared with both stimulant-dependent individuals (SDIs; *n*=42) and controls (*n*=47), whereas SDIs and controls did not differ from one another in activation in this region. (**a**) Group differences in functional magnetic resonance imaging (fMRI) activation in the left IFG, representing a decrease in activation in the siblings. Error bars represent one standard error of the mean; covariates include gender, smoking status, Alcohol Use Disorder Identification Test (AUDIT) and BDI) scores. (**b**) fMRI general linear model between group contrast activation with application of the IFG mask. A significant contrast cluster emerged in the left IFG.

After restriction of the analysis to the IFG, an additional cluster was revealed in the left hemisphere that significantly differed between groups during the incongruent–congruent contrast (F(2,135)=11.981, *P*<0.001). The siblings again had significantly lower activity than both SDIs (*P*<0.001) and controls (*P*=0.006), whereas SDIs and controls did not significantly differ (Figure 2 and Table 2). Application of a mask of the ACC did not result in any significant clusters emerging that differed in activation in this region between the three groups.

### Regression of Stroop performance to neuroimaging findings

When behavioral interference scores were regressed onto the incongruent–congruent imaging contrast in CamBA, no significant areas were found. However, upon application of the IFG mask, two bilateral clusters arose that significantly deactivated in association with interference scores among all participants.

Follow-up analyses in SPSS confirmed that activity in these areas negatively correlated with behavioral performance in all participants (left: *r*=−0.351, *P*<0.001; right: *r*=−0.398, *P*<0.001), with greater deactivation during the contrast signifying greater interference. When each group was analyzed individually, the correlation with interference scores during the incongruent–congruent interference regression contrast remained significant in the left (*r*=−0.454, *P*=0.001) but not right IFG for control participants. Among SDIs, interference scores negatively correlated with activity in both the left and right IFG during the incongruent–congruent interference regression contrast (left: *r*=−0.366, *P*=0.017; right: *r*=−0.580, *P*<0.001). In sibling participants, only right IFG activation in the incongruent–congruent interference regression contrast negatively correlated with interference scores (*r*=−0.377, *P*=0.008).

### Relationship of structural changes to Stroop performance

**Figure 3:**
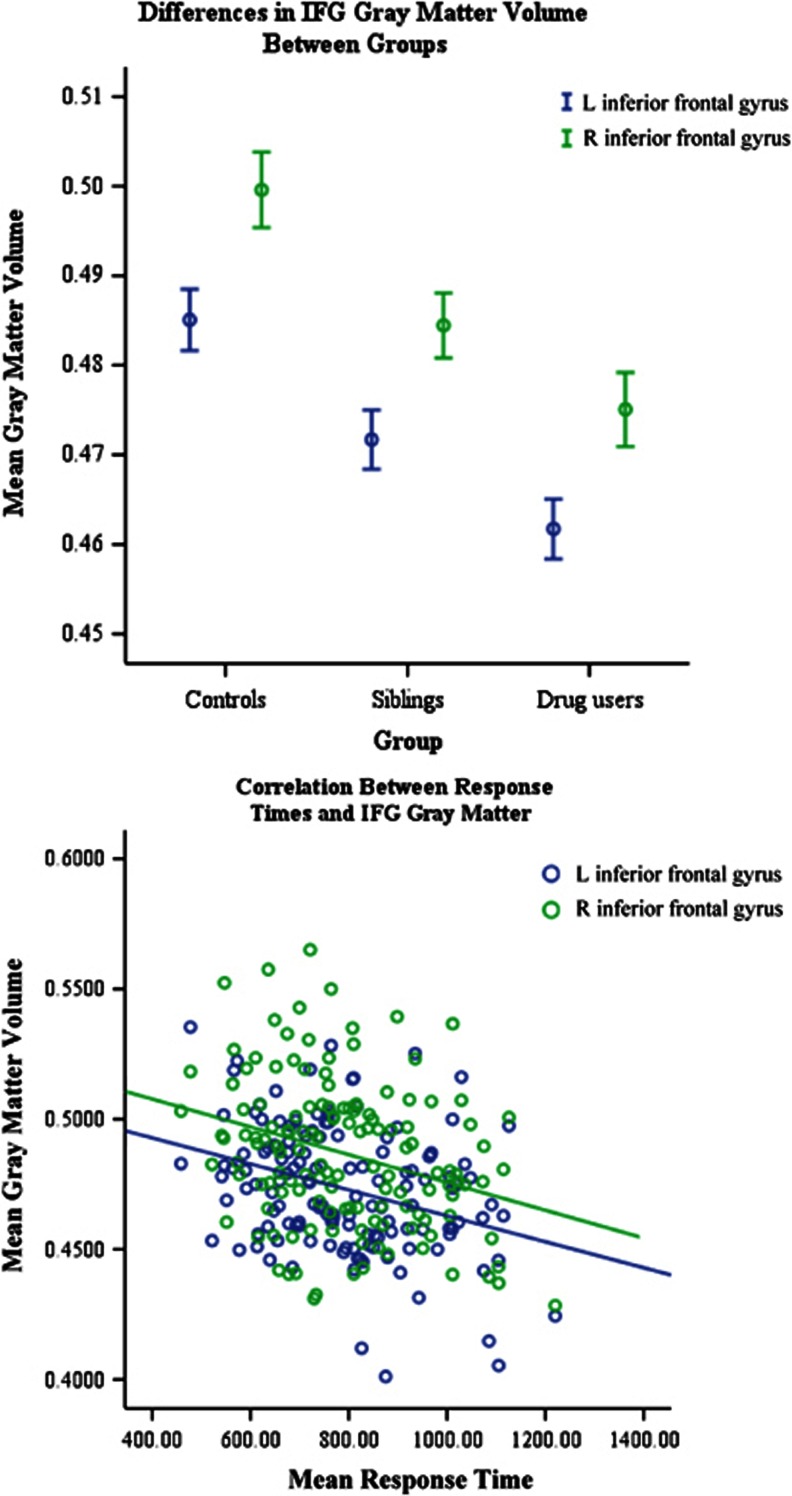
Structural differences in the inferior frontal gyrus (IFG) between stimulant-dependent individuals (SDIs; *n*=42), their siblings (*n*=49) and controls (*n*=47). (**a**) SDIs and siblings both demonstrated a significant decrease in gray matter volume in the bilateral IFG as compared with control individuals. There was no difference in volume between SDIs and their siblings. Error bars represent 1 s.e.m.; covariates include gender, smoking status, Alcohol Use Disorder Identification Test (AUDIT) and Beck Depression Inventory (BDI) scores. (**b**) IFG GM volume decreases significantly correlated bilaterally with an increase in response times on both conditions of the color Stroop task, with a decrease in volume relating to an increase in response latencies.

A structural analysis comparing GM volume between these groups revealed further differences in performance based on cortical volume. Upon application of the IFG mask onto the voxel-based morphometry analysis, we discovered differences in GM volume bilaterally in the IFG in both SDIs and their siblings as compared with controls (left: F(2,135)=11.749, *P*<0.001; right: F(2,135)=9.384, *P*<0.001). These structural changes related to behavioral performance, such that bilateral decreases in IFG GM correlated with RTs on both congruent and incongruent trials among all participants (congruent: left *r*=−0.337, *P*<0.001; right *r*=−0.320, *P*<0.001; incongruent: left: *r*=−0.253, *P*=0.003; right *r*=−0.224, *P*=0.008; Figure 3). However, interference scores did not correlate with cortical volume. In addition, IFG volume and activity levels did not correlate.

### Effects of drug use history on performance

Among the SDIs, there was a significant correlation between years of use and errors made (*r*=0.388, *P*=0.011), such that the longer an individual had used stimulants, the greater the number of errors were committed. There were no other significant relationships between drug use history and either color-word Stroop performance or neuroimaging activation. This includes time since last stimulant use, which did not correlate with any measure of task ability, including reaction times, interference scores and errors committed, or functional activation on the Stroop. A median split for time since last use (⩾2 days) also did not reveal any significant differences in either behavioral or functional performance.

## Discussion

SDIs and their non-dependent siblings were significantly more impaired on the color-word Stroop than unrelated healthy controls, as demonstrated by longer response latencies during congruent trials for SDIs and incongruent trials for their siblings. However, the two groups did not differ from one another on any variable, and there were no differences between groups in interference scores. In neural terms, significant differences were evident between the siblings and both stimulant-dependent and control participants, with siblings comparatively underactivating the bilateral IFG and left superior/middle frontal gyrus. However, the SDIs and controls did not differ in the activity levels in any region. Two primary questions arise from these results: why was there no difference in activity between SDIs and controls despite differences in behavioral performance? and why did the siblings significantly underactivate frontal gyral regions compared with the other groups?

The lack of differentiation between groups on interference scores suggests that, despite slowing in the SDIs and siblings, there was no greater dysfunction on the challenging incongruent condition. Instead, there appeared to be globalized slowing or inefficiency, rather than task-specific impairment. This absence of differences in interference scores was not unexpected, as previous studies investigating color-word Stroop performance in SDIs have shown no impairments compared with controls.^23, 24, 25, 26^ Cognitive slowing corresponded to structural differences in the IFG in both SDIs and their siblings, with decreases in GM volume correlating with increased RTs on both conditions. This suggests these structural changes affect cognitive efficiency, but do not cause a condition-specific impairment. Earlier findings in these groups support these results, with decreases in white matter connectivity adjacent to the IFG associated with increased slowing on a motor control task.^14^

Among all participants, a decrease in IFG activity was associated with impaired performance via increased interference scores. However, within each group a different pattern of activation emerged, indicating hemispheric differences between control, sibling and stimulant-dependent participants. Activation in the controls adhered to the standard findings for Stroop performance, with decreases in left IFG activity associated with increased interference scores.^16^ However, in the SDIs bilateral IFG activations negatively correlated with performance, and in the siblings only right hemispheric activation was related to task interference, with decreased right IFG activity correlating with higher interference scores.

We theorize that the decrease in bilateral IFG GM in the stimulant-dependent and sibling participant groups contributed to this disproportionate right hemisphere recruitment, an area not typically activated during the Stroop due to the task's left-lateralized semantic load and right-handed motor response. The controls did not show this right IFG response, instead deactivating the region, presumably to avoid interfering activity and enabling more efficient processing. Indeed, an increase in right IFG activation among all participants was linked to greater impairment on the task, compensatory activity potentially leading to decreased cortical efficiency and subsequent increases in interference. However, we do caution that these correlations were exploratory in nature, and the resulting explanation is only one possible reading of the data; thus, it should be taken with care. We have made a tentative interpretation of the data, but these results need to be followed up with future research into cortical inefficiency and a loss of laterality in drug users and their siblings.

Prefrontal contralateral compensation has previously been observed in drug users on a decision-making task, with current and prior users experiencing an increase in left orbitofrontal cortex activation, whereas controls activated the right dorsolateral prefrontal cortex.^44^ This loss of laterality has also been seen in stimulant users on a finger tapping task, recruiting bilaterally from cortical and subcortical areas.^45^ However, this is the first study to report such findings in the non-dependent siblings of SDIs, suggesting an endophenotype of decreased structural integrity and compensatory contralateral activation.

The question remains as to why SDIs, despite their similar patterns of activation, had such a significant increase in activity compared with their siblings. It is possible that the hypoactivation exhibited by the siblings was the endophenotype-like response, and that SDIs, before drug abuse, demonstrated similar decreases in activation during inhibitory control. However, the effect of stimulant use, whether chronic or acute, may have altered this original response. A recent assessment of motor inhibition in adolescents showed similar results, with a relative overactivation in the same right IFG region in adolescents who had experimented with drugs compared with those who had not.^46^ This suggests that the increase in neural activation during cognitive control (including inhibitory response) occurs relatively early as a consequence of drug abuse. Conversely, the decreased activation in the siblings could be representative of a protective factor against drug abuse, despite similar manifestations of behavioral impairments in inhibitory control. This account suggests that SDIs and siblings may experience similar baseline activation, but the effects of stimulants elevated SDIs' blood oxygen level-dependent activity to those of controls.

Alternatively, as 93% of SDIs tested positive for stimulants, their relative hyperactivation could be an acute effect of stimulants on the brain. Cocaine can increase neural activity,^47^ elevating low baseline levels in abstinent users to those of non-drug-using controls.^48^ This effect has been proposed to support the self-medication hypothesis in individuals with decreased dopaminergic activity. However, time since last use did not correlate with any behavioral or functional imaging results in the SDIs. Moreover, a median split conducted on time since last use did not reveal any significant differences in performance or activation between individuals with shorter or longer periods of abstinence. Thus, we do not believe that either the acute effect of stimulants or stimulant withdrawal significantly affected SDIs' behavioral performance or functional activation on the task.

The absence of activation in the ACC, a key area in Stroop performance,^16, 18, 20, 23, 49^ was perhaps surprising. However, absence of cingulate activation on the Stroop has also been noted in other studies with drug-using participants.^24, 31^ The ACC is thought to control conflict monitoring, and it is most activated during changing task demands (such as switching from congruent to incongruent stimuli), resulting in greater cognitive conflict.^49^ However, this study used a block design, circumventing the change in task demands. This adjustment of task structure may explain the lack of ACC activation, as there was no commonly cited conflict, with incorrect responses requiring behavioral adjustments on subsequent trials.^49^ As only correct responses were included in this model, it is possible that this region was not significantly recruited during correct responding. Finally, dissociation between IFG and ACC activation during the Stroop has been suggested, the ACC compensating for diminished IFG control.^16^ Given the significant increase in IFG activity, particularly in SDIs and controls, as well as the contralateral compensatory activation in siblings and SDIs, it is possible that the ACC was not requisitely recruited by participants during the current task.

Weaknesses of the study include a disparate number of participants in each group. Use of an event-related rather than block design might also have produced more robust findings, particularly in the anterior cingulate. Subsequent studies could employ genetic analysis to elucidate whether similarities in performance are due to inherited traits or shared environmental experiences between the sibling and stimulant-dependent groups. As the sibling pairs were raised in the same households, it is difficult to discern the extent to which environmental effects, such as low socioeconomic status, affected these individuals. These experiences could cause them to differ in performance from control participants, who were less likely to be exposed to these challenges growing up. Also, as the SDIs had significantly higher levels of depression and alcohol use, it would be ideal to control for these factors during recruitment. Finally, the relatively short abstinence period of the SDIs should be better controlled for, with a minimum abstinence requirement to ensure results are not effects of either acute stimulant use or withdrawal, but are stable traits of this group.

### Summary

This study investigated cognitive control and correlative neural activation in SDIs, their non-dependent siblings and unrelated healthy controls. A decline in efficiency, relating to impairments, does not result from drug abuse, but are instead underlying risk factors for addiction. In addition, impairment in hemispheric lateralization during task performance was apparent in both groups, indicating a potential compensatory increase in activation in contralateral regions. However, there was a significant difference between SDIs and their siblings in neural activation, the siblings underactivating relevant regions, while the SDIs had a relative increase in activity, particularly in the IFG. This important difference may possibly be attributed to the effects of stimulants on brain activation when performing this cognitive control task.

## Figures and Tables

**Table 1:** Demographic and baseline characteristics for 42 SDIs, 49 of their biological siblings and 47 healthy control volunteers, and mean behavioral results on the Stroop task for each of the three participant groups

	*Control (*n*=47), mean (s.d.)*	*Sibling (*n*=49), mean (s.d.)*	*SDI (*n*=42), mean (s.d.)*	*F/*χ*2*	P*-value*
*Demographics*					
Age	32.34 (8.63)	32.63 (8.35)	34.24 (7.39)	0.684	0.506
Sex (% male)	63.8%	49.0%	95.2%	21.45	<0.001
IQ (NART)	112.67 (8.09)	108.91 (8.88)	110.64 (7.46)	2.42	0.093
Education (years)	12.70 (1.92)	12.12 (2.00)	11.69 (1.70)	3.22	0.043
Depression (BDI-II score)	2.21 (2.56)	5.22 (6.19)	18.43 (12.18)	53.45	<0.001
Smoking status (% smoker)	10.6%	55.1%	92.9%	68.16	<0.001
Average daily cigarettes	2.47 (4.79)	5.08 (7.82)	15.92 (13.02)	17.50	<0.001
Alcohol use (AUDIT score)	3.32 (2.28)	3.86 (4.50)	12.51 (11.53)	23.53	<0.001
					
*Drug use*					
DAST-20	0.00 (0.00)	0.51 (1.10)		−3.18	0.002
OCDUS			23.86 (9.07)		
Age onset stimulant use			16.45 (2.86)		
Years stimulant use			15.74 (6.44)		
Urine stimulants (%)			92.9%		
Last stimulant use (days)			2.17 (2.41)		
					
*Behavioral Stroop results*					
Congruent med RT (ms)	653.17 (125.99)	723.14 (137.50)	768.99 (171.36)	7.24	0.001
Incongruent med RT (ms)	802.72 (181.25)	904.85 (214.90)	910.76 (188.10)	4.45	0.013
Interference med RT (ms)	149.55 (140.08)	181.70 (173.58)	141.77 (171.05)	0.797	0.454
Total mean errors (%)	2.30 (7.2%)	3.37 (10.5%)	3.43 (10.7%)	1.92	0.151

Abbreviations: AUDIT, Alcohol Use Disorder Identification Test; BDI-II, Beck Depression Inventory; DAST-20, Drug Abuse Screening Test; NART, National Adult Reading Test; OCDUS, Obsessive Compulsive Drug Use Scale; RT, response time; SDI, stimulant-dependent individuals.

Congruent and incongruent trial results are represented via median response latencies, interference scores are reported as the difference in response times between the two trial conditions and total mean errors for each group are given. Behavioral results were corrected for gender, smoking status, AUDIT and BDI scores.

**Table 2:** Mean activation cluster voxels for each imaging contrast among all groups

*Contrast activation areas*	*Broadmann area*	*Cluster size (voxels)*	*Peak value coordinates*
*Whole-brain contrast (incongruent–congruent) analysis among all participants*			
* *Cluster 1			
* *Right rolandic operculum, right superior temporal gyrus, right heschl gyrus, right insula, right postcentral gyrus, right supramarginal gyrus	13, 22, 40, 41, 42, 43	105	60, −22, 12
* *Cluster 2			
* *R caudate	125	59	4, 4, 12
Cluster 3			
* *Left inferior frontal gyrus—triangularis, left inferior frontal gyrus—opercularis, left precentral gyrus	9, 45, 46	114	−50, 14, 28
* *Cluster 4			
* *Left precentral gyrus, left middle frontal gyrus, left postcentral gyrus	6, 8, 9	126	−44, 4, 34
			
*Between-group contrast comparison*			
* *Cluster 1			
* *Right rolandic operculum, right insula, right caudate, right supramarginal gyrus	13	232	40, −26, 28
* *Cluster *2*			
* *Left medial superior frontal gyrus, left middle frontal gyrus, left superior frontal gyrus, left inferior frontal gyrus—opercularis	8, 24, 32	110	−28, 14, 34
			
*Between-group comparison with IFG mask*			
* *Cluster 1			
* *Left inferior frontal gyrus—opercularis, left inferior frontal gyrus—triangularis, left rolandic operculum, left insula	13	43	−42, 10, 20
			
*Interference regression activation with IFG Mask*			
* *Cluster 1			
* *Left inferior frontal gyrus—triangularis, left insula	13, 45, 47	25	−38, 28, 4
* *Cluster 2			
* *Right inferior frontal gyrus—triangularis, right inferior frontal gyrus—operculum	13, 45	30	42, 24, 12

Abbreviations: IFG, inferior frontal gyrus; MNI, Montreal Neurological Institute.

Contrasts represent activation on incongruent compared with congruent trials, first among all participants and then comparing contrast activations between groups. Interference scores (differences between incongruent and congruent response times) are regressed onto contrast activations in the fourth analysis, using all participants. Significance set a *P*<0.05 family-wise error correction for multiple comparisons. Coordinates listed are in MNI standard space.
