# Supplementary material for: Cognitive control dysfunction and abnormal frontal cortex activation in stimulant drug users and their biological siblings
Source: Transl Psychiatry. 2013 May 14;3(5):e257–. doi: 10.1038/tp.2013.32 (PMC3669919; doi:10.1038/tp.2013.32)
Supplement: Supplementary Information [file tp201332x1.pdf]

Supplemental Information

S1: Demographic group post-hoc comparisons

Demographic variable	ANOVA		Bonferroni post-hoc *P*-value		
	F	*P*	Control vs. SDI	Control vs. Sibling	SDIs vs. Sibling
Age	.684	.506	.828	1.00	1.00
BDI	53.45	<.001*	<.001*	.183	<.001*
Education	3.22	.043*	.039	.406	.837
NART	2.42	.093	.770	.089	1.00
AUDIT	23.53	<.001	<.001*	1.00	<.001*
	χ^2		*P*-value		
Sex	21.45	<.001*	<.001*	.205	<.001*
Smoking status	68.16	<.001*	<.001*	<.001*	<.001*

* Significant at $p<.05$

S2: As sex, education, smoking status, depression scores and alcohol use all significantly differed between participant groups, largely driven by the stimulant-dependent individuals, we included these variables as covariates in the general linear model multivariate regression analysis. However, there were no differences in the results both with and without these covariates. As these variables seem to be somewhat intrinsically linked to drug addiction, nicotine dependence, alcohol abuse and depression commonly occurring as co-morbidities in the disorder, we thought it most prudent to report the results without using these variables as covariates (1). However, we report the results with covariates included in the model here as there were slight discrepancies.

	Covariates included		Covariates excluded	
Behavioral analysis	*F*(2,129)	*P*	*F*(2,135)	*P*
Congruent RT	3.738	.026	7.243	.001
Incongruent RT	3.857	.024	4.452	.013
Interference	.955	.387	.797	.45
Errors	2.923	.057	1.915	.151
Imaging analysis: *First level*				
Left precentral / middle frontal gyrus	.136	.873	.330	.719
Left inferior frontal gyrus	.735	.482	.261	.771
Right caudate	.864	.424	.542	.583
Right rolandic operculum	.162	.851	.001	.999
Behavioral analysis: *Second level*				
Right insula / rolandic operculum	16.936	<.001	18.373	<.001
Left medial superior frontal gyrus	11.854	<.001	12.094	<.001
Behavioral analysis: *Second level*: Inferior frontal gyrus mask				
Left inferior frontal gyrus	8.955	<.001	11.981	<.001

Covariates in the model include gender, years of education, smoking status, AUDIT alcohol use scores and BDI depression scores.

1. Miller GA, Chapman JP. Misunderstanding analysis of covariance. *J Abnorm Psychol* 2001; **110**(1): 40-8.
